# Gastric cancer and genomics: review of literature

**DOI:** 10.1007/s00535-022-01879-3

**Published:** 2022-06-25

**Authors:** Takumi Onoyama, Shumpei Ishikawa, Hajime Isomoto

**Affiliations:** 1 Department of Preventive Medicine, Graduate School of Medicine, The University of Tokyo, 7-3-1 Hongo, Bunkyo-ku, Tokyo, 113-0033 Japan; 2 Division of Gastroenterology and Nephrology, Department of Multidisciplinary Internal Medicine, Tottori University Faculty of Medicine, 36-1 Nishi-cho, Yonago, Tottori 683-8504 Japan

**Keywords:** Gastric cancer, Genomics, Next-generation sequence, Liquid biopsy, Precision medicine

## Abstract

Gastric cancer (GC) is a major health concern in many countries. GC is a heterogeneous disease stratified by histopathological differences. However, these variations are not used to determine GC management. Next-generation sequencing (NGS) technologies have become widely used, and cancer genomic analysis has recently revealed the relationships between various malignant tumors and genomic information. In 2014, studies using whole-exome sequencing (WES) and whole-genome sequencing (WGS) for GC revealed the entire structure of GC genomics. Genomics with NGS has been used to identify new therapeutic targets for GC. Moreover, personalized medicine to provide specific therapy for targets based on multiplex gene panel testing of tumor tissues has become of clinical use. Recently, immune checkpoint inhibitors (ICIs) have been used for GC treatment; however, their response rates are limited. To predict the anti-tumor effects of ICIs for GC and to select patients suitable for ICI treatment, genomics also provides informative data not only of tumors but also of tumor microenvironments, such as tumor-infiltrating lymphocytes. In therapeutic strategies for unresectable or recurrent malignant tumors, the target is not only the primary lesion but also metastatic lesions, and metastatic lesions are often resistant to chemotherapy. Unlike colorectal carcinoma, there is a heterogeneous status of genetic variants between the primary and metastatic lesions in GC. Liquid biopsy analysis is also helpful for predicting the genomic status of both primary and metastatic lesions. Genomics has become an indispensable tool for GC treatment and is expected to be further developed in the future.

## Introduction

Gastric cancer (GC), with an incidence of more than 1 million per year worldwide, is a major health issue in many countries, with a high prevalence in Asia, Africa, South America, and eastern Europe. More than 700,000 patients with GC have died worldwide [1, 2].

GC is a heterogeneous disease stratified by histopathological variants. The histopathological classification proposed by Lauren, classifying GCs into “intestinal” and “diffuse” subtypes, is most widely used. Intestinal type GC is associated with intestinal metaplasia caused mainly by *Helicobacter pylori* infection and has tubular or glandular structures, while diffuse-type GC typically comprises of poorly differentiated tumor cells that lack adhesion and infiltrate the stroma as single cells or small subgroups. Although these histopathological differences are associated with GC prognosis, histopathological variations are not routinely used as the basis for determining GC treatment and management.

**Fig. 1:**
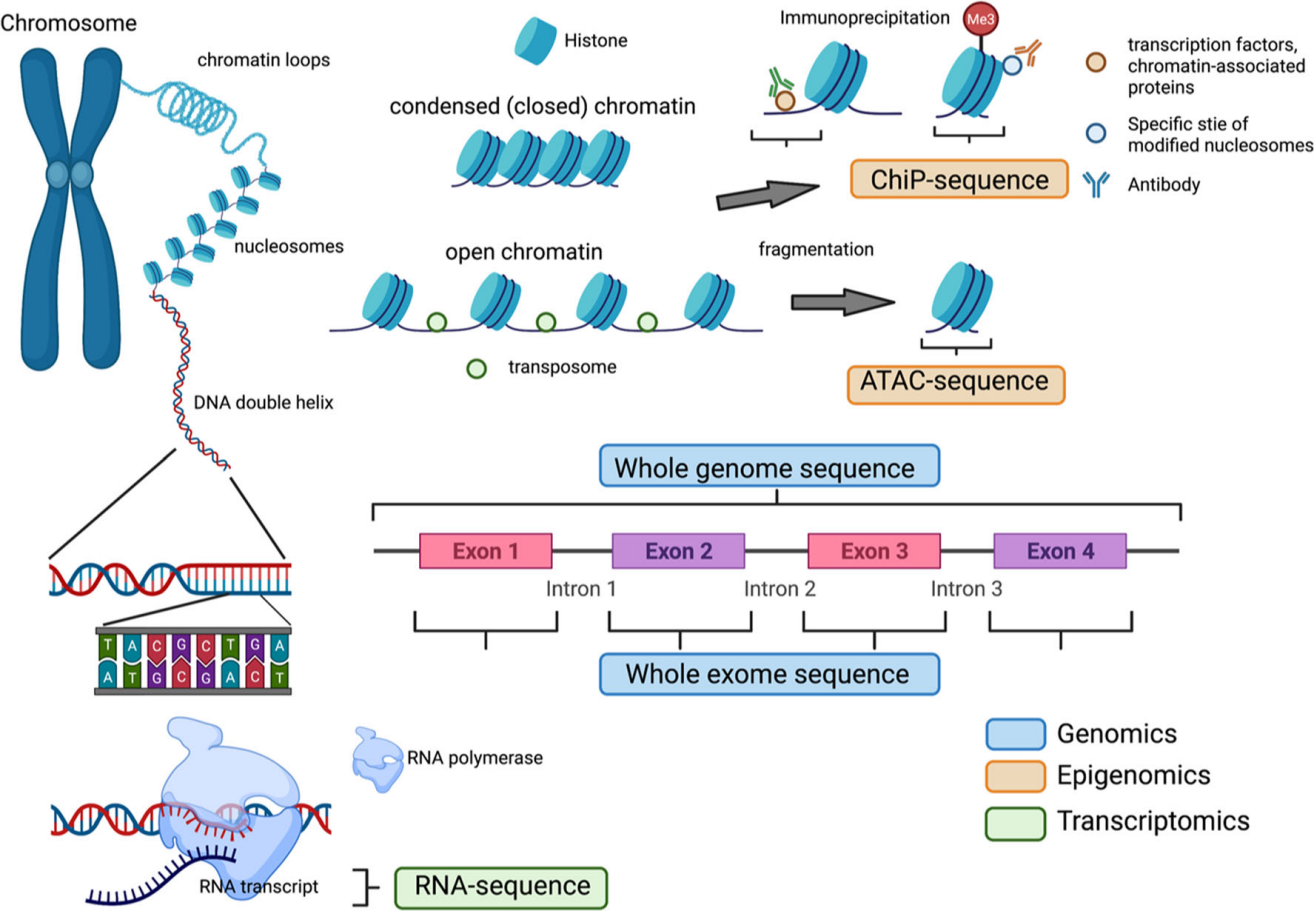
Genomics, epigenomics, and transcriptomics with next-generation sequencing. In genomics, the whole-exome sequence and whole-genome sequence, reading every single base of DNA systematically, can reveal copy number variations, single nucleotide variants, and gene-fusion events in the whole exome or whole genome. In epigenomics, chromatin immunoprecipitation-sequencing is used to assay the DNA fragments that bind to these transcription factors, other chromatin-associated proteins, or sites that correspond to modified nucleosomes specifically selected using antibodies. The assay for transposase-accessible chromatin using sequencing is an epigenomics technique that captures open chromatin sites using transposase, which can be inserted only in regions of open chromatin, revealing the interplay between genomic locations of open chromatin, DNA-binding proteins, individual nucleosomes, and chromatin compaction at nucleotide resolution. RNA sequencing (RNA-seq), a transcriptomics technique, can not only read every single base of RNA fragment or complementary DNA from large amounts of transcriptome data, but can also be classified into three types, including exonic reads, junction reads, and poly(A) end-reads, assemble transcriptomes with or without a reference genome, and evaluate gene expression profiling

Next-generation sequence (NGS) technologies have become widely used in genomics, epigenomics, transcriptomics, etc. In genomics, whole-exome sequencing (WES) and whole-genome sequencing (WGS), which systematically reads every single base of DNA, can reveal copy number variations, single nucleotide variants (SNV), and gene-fusion events in the whole exome or whole genome [3, 4]. RNA sequencing (RNA-seq), one of the transcriptomics, could not only read every single base of RNA fragment or complementary DNA (cDNA) from large amounts of transcriptome data, but can also classify several subtypes including exonic reads, junction reads, poly(A) end-reads, and non-cording RNA; assemble transcriptome with or without reference genome; and evaluate gene expression profiling [5–7]. In epigenomics, chromatin immunoprecipitation (ChIP)-sequencing is used to assay the DNA fragments that bind to these transcription factors, other chromatin-associated proteins (i.e., non-histone ChIP), or sites that correspond to modified nucleosomes (i.e., histone ChIP) specifically selected using antibodies [8]. The assay for transposase-accessible chromatin using sequencing (ATAC-seq) is an epigenomics technique that captures open chromatin sites using transposase, which can be inserted only in regions of open chromatin, revealing the interplay between genomic locations of open chromatin, DNA-binding proteins, individual nucleosomes, and chromatin compaction at nucleotide resolution [9] (Fig. 1). Developments in cancer genomic, epigenomic, and transcriptomic analyses using NGS have recently revealed relationships between various malignant tumors and genomic information. In 2014, some studies of the WES and WGS for GC were also reported, and the entire structure of GC genomics was revealed [10–12]. These studies will help identify new therapeutic targets for malignant diseases. Moreover, some existing therapeutic agents, including chemotherapy, molecularly targeted therapies, and immune checkpoint inhibitors (ICIs), have been selected for the appropriate patient population. In clinical practice, personalized medicine, based on multiplex gene panel testing for tumor tissues obtained from individual patients, has been put to practical use. These genomic data will be informative for determining GC treatment and be seen as being as instructive as clinical staging, including the extent of primary tumor invasion, regional lymph node involvement, and presence of metastatic spread.

In this review, we highlight the basic and clinical advances in GC genomics and focus on the direct clinical actionability of genomic targets and treatment policies.

## Molecular classification of gastric cancer

**Fig. 2:**
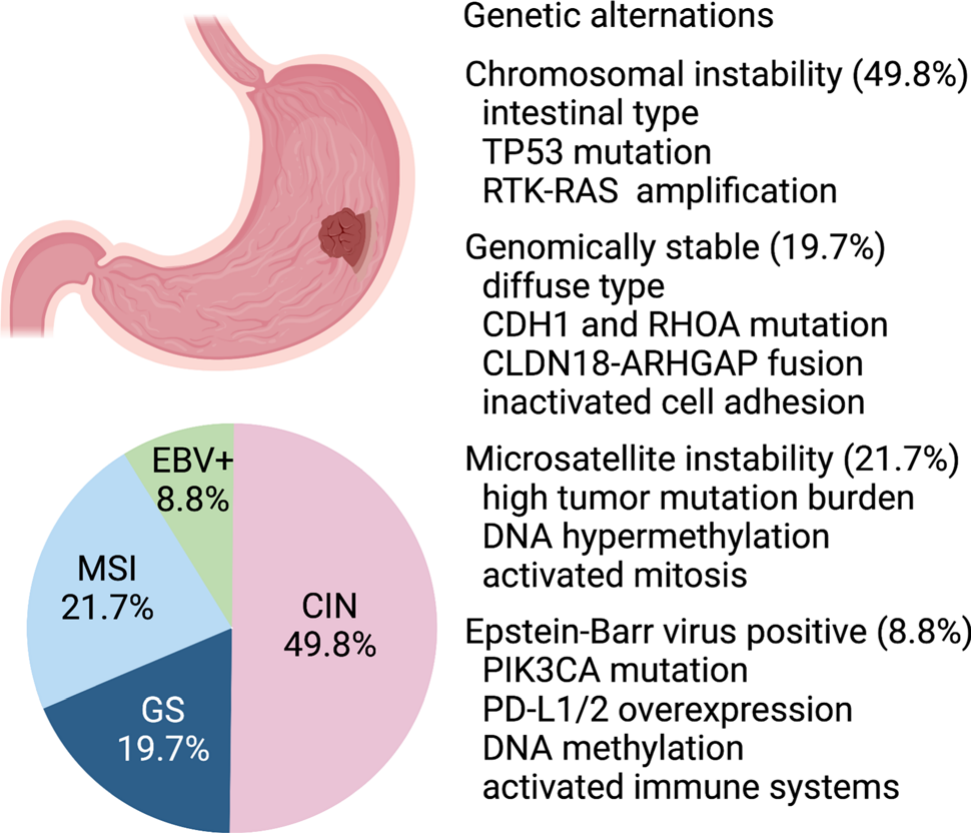
Molecular subtypes of gastric cancer classified via genomics. Gastric cancer is classified into four molecular subtypes: chromosomal instability (CIN), genome stability (GS), microsatellite instability (MSI), and Epstein-Barr virus (EBV) positivity. Most subtypes of CIN correspond to the intestinal type and are accompanied by *TP53* mutations and tyrosine kinase receptor-*RAS* signal amplification. Most GS subtypes correspond to the diffuse type according to the histopathological classification of GC. In MSI- and EBV-positive subtypes, *phosphatidylinositol-4,5-bisphosphate 3-kinase catalytic subunit alpha* (*PIK3CA*) and *AT-rich interaction domain 1A (ARID1A)* gene mutations are enriched. Furthermore, programmed cell death ligand 1/2 (PD-L1/2) is often overexpressed via gene amplification and structural variation in EBV-positive subgroups

The cancer genome atlas (TCGA) and the Asian cancer research group classified GC into 4 molecular subtypes: chromosomal instability (CIN), genome stability (GS), microsatellite instability (MSI), and Epstein–Barr virus (EBV) positivity as DNA-based alterations [12, 13] (Fig. 2). Some characteristic genomic abnormalities of each of the four subtypes have been clarified, which will be helpful in identifying new therapeutic targets in GC. Most subtypes of CIN correspond to the intestinal type, whereas most subtype GS correspond to the diffuse type in the histopathological classification of GC. Tyrosine kinase receptor amplifications are often identified in subtype CIN, and *phosphatidylinositol-4,5-bisphosphate 3-kinase catalytic subunit alpha (PIK3CA)* and *AT-rich interaction domain 1A (ARID1A)* gene mutations are enriched in subtype MSI and EBV positivity. Research and clinical trials targeting these kinases are progressing, and new treatments may appear in the future. In addition, *programmed cell death ligand 1/2 (PD-L1/2)* is often overexpressed via gene amplification and structural variation in EBV-positive subgroups, and ICIs can be effective. In subtype MSI, with high-frequency gene mutations and neoantigen expression, ICIs are also effective [14]. Meanwhile, few genomic abnormalities, including chromosome amplification and deletion, are found in the GS subtype compared with the others. Therefore, ICIs are thought to be ineffective for the GS subtypes. Indeed, among 61 patients with metastatic GC, the efficacy of ICIs for subtype GS was only 12%, whereas those for subtype MSI and EBV positivity were 85.7 and 100.0%, respectively [14]. Furthermore, the expression of *human epidermal growth factor receptor 2 (HER2)*, which can be a therapeutic target, was low in the GS subtype. Therefore, advanced approaches are needed to improve the prognosis of the GS subtypes.

## Risk for gastric cancer including germline mutations and somatic variants

Hereditary diffuse GC (HDGC), described in 1998, is a cancer syndrome characterized by a high prevalence of diffuse-type GC and breast cancer with autosomal dominant inheritance [15]. Most confirmed HDGC cases are caused by inactivating germline mutations in *cadherin 1 (CDH1)* encoding E-cadherin, whereas a small minority of HDGC cases are caused by mutations in *α-catenin (CTNNA1)* [16]. In addition, WESs for HDGC patients without *CDH1* variants revealed some germline mutations, such as *BRCA1/2*, *partner and localizer of BRCA2 (PALB2)*, and *RAD51* mutations [17, 18]. However, HDGC is a small part of the whole GC, and inherited genetic variants contribute to < 3% of GC [19]. In general, GC develops not only due to genetic factors, but also environmental factors, such as *Helicobacter pylori* infections. Therefore, it is difficult to accurately estimate the association between GC and various genetic factors.

In 2020, WES for 243 Japanese participants with GC was reported and revealed germline *CDH1* variants, most of which were shown in diffuse-type GC and were attributed to a few recurrent SNV shared by the Japanese and Koreans [20]. This variant, shown in 13.3% (14/105 participants) of diffuse-type GC, consists of 5 SNVs, and 4 of them were included in the germline variant related to HDGC. However, the variant was not necessarily related to a family history of GC, and half of the participants were regarded as sporadic cases. Although the frequency of the rare germline *CDH1* variant was 3.4%, this might be an important information for detecting the high-risk GC population. The study also performed WES for comparing the GC exome profiles of 319 Asian patients to 212 non-Asian patients and revealed the distinct GC subclass with alcohol-associated mutation signature and Asian-specific defective aldehyde dehydrogenase 2 family member allele [20]. Genomics is also useful for detecting populations at risk for GC.

## Genomics for diffuse-type gastric cancer and possible therapeutic targets for somatic mutations

Most diffuse-type GC, known as the histopathological classification of GC with poor prognosis, corresponds to the GS subgroup in the molecular subtypes via genomic analysis and has low-frequency structural chromosome aberration and gene mutations that could be targets for treatment [12]. Therefore, many studies on the clarification of diffuse-type GC have been performed using various genomics approaches. Some characteristic genetic variants, such as *CDH1* germline mutations and *Ras homolog family member A (RHOA)* driver gene mutations, are known to be part of the GS subtype. Germline mutations of *CDH1*, cording E-cadherin (one of the cell adhesion molecules), do not seem to be a target of treatment because that is the loss-of-function mutation. Meanwhile, *RHOA* signal mutations, newly identified as a driver gene in subtype GS, are expected to become a therapeutic target because *RHOA* gene mutations, where there are some hotspots, such as Y42C, R5W, G17E, L57V, and L69R, are known as gain-of-function mutations [11, 21]. Therefore, it may be possible to become a target of treatment, although the downstream of *RHOA* is still controversial [21, 22]. However, *RHOA* is a guanosine triphosphate (GTP)-binding protein; therefore, it is difficult to develop inhibitors, such as *KRAS* [11, 23].

In addition, structural abnormalities, such as fusion genes, can be detected by whole-genome or whole-transcriptome analysis. In diffuse-type GC, multiple *claudin 18 (CLDN18)-rho GTPase activating protein (ARHGAPs)* fusion genes have been reported, and since *ARHGAP* exists in the same pathway as *RHOA*, it may be associated with both variants of *RHOA* and abnormalities in the *RHOA* signaling pathway [24]. In a report that analyzed 32 cases of gastric signet ring cell carcinoma, the *CLDN18-ARGAP26/6* fusion gene was found in eight cases (25%) [25]. Among additional 797 participants with gastric signet ring cell carcinoma, patients with the *CLDN18-ARGAP26/6* fusion gene were younger than patients without the *CLDN18-ARGAP26/6* fusion gene (mean, 51.3 years old vs 60.7 years old), and that fusion was more frequent among females than males (18.5 vs. 4.6%). Unfortunately, there was no significant difference between the prognosis of patients who received chemotherapy and those who did not among GC patients with the *CLDN18-ARGAP26/6* fusion gene. The other genetic analysis for participants with diffuse-type GC also showed that the *CLDN18-ARGAP* fusion gene was enriched in the younger population and was related to a significantly poorer prognosis compared to subgroups without the *CLDN18-ARGAP* fusion [26, 27]. Considering these facts, the present chemotherapies may not be effective for GC with the *CLDN18-ARGAP26/6* fusion gene. CLDN18.2, an epithelial tight junction protein, is strictly confined to tight junctions in the gastric mucosa. However, in GC, perturbations in cell polarity lead to CLDN18.2 being exposed on the cancer cell surface and targetable by antibodies [28, 29]. AMG 910, an antibody construct designed to engage CD3-positive T cells with CLDN18.2-expressing GC cells, whose anti-tumor effect for CLDN18.2-positive GC or gastroesophageal junction (GEJ) adenocarcinoma is now studied, is expected to be effective [30].

Kumagai et al. reported the tumor microenvironment (TME) of GC with *RHOA* abnormalities [31]. Genomic analysis and flow cytometry for tumor-infiltrating lymphocytes (TILs) revealed a subset with low expression of genes associated with immune response and high-frequency infiltration of regulatory T lymphocytes (Treg), and almost half of the subset had *RHOA* Y42C mutation. *RHOA* mutation in cancer cells activated the *phosphoinositide-3-kinase (PI3K)-AKT*-*mammalian target of rapamycin (mTOR)* signaling pathway and reduced the C–X–C motif chemokine ligand (CXCL)10/CXCL11, recruiting effectors of CD8 + T lymphocytes. Moreover, the fatty acid synthase gene was upregulated in *RHOA* Y42C-mutated GC cells compared to *RHOA* wild-type GC cells, indicating that the intake of fatty acids synthesized via Tregs was upregulated. They also showed the efficacy of anti-programmed cell death-1 (PD-1) antibody and PI3K inhibitor in mouse xenograft models with newly implanted *RHOA* Y42C-mutated GC strains, indicating that the combination of anti-PD-1 antibody and PI3K inhibitor was more effective than anti-PD-1 antibody alone. Therefore, in *RHOA* Y42C-mutated GC, high-frequency infiltration of Treg cells and the immune response to GC were downregulated. One possibility, in the future, is that combination therapy of anti-PD-1 antibody and PI3K inhibitor might improve the therapeutic effect for *RHOA* Y42C-mutated GC patients. Recently, multi-omics for malignant ascites of GC revealed that disseminated GC was stratified into 2 groups, and 1 of them, with active EMT according to the expression profile, bearing *transforming growth factor-β* pathway activation through SMAD3 SE activation and high expression of the transcriptional enhancer factor TEF-1 [32]. Inhibition of the *TEAD* pathway circumvents resistance to therapy, suggesting a potential molecular-guided therapeutic strategy. In TCGA reports, subtype GS is known to be overlapped with diffuse-type GC, but it is not complete, with 29 (42.0%) of diffuse-type GC in the TCGA cohort not being subtype GS [12]. Although all the cases examined in Tanaka et al. have intra-abdominal metastases and malignant ascites, characteristics of advanced diffuse-type GC, with 50 (52.1%) of *CDH1* variant and 15 (15.3%) of *RHOA* variant. However, they had several features that were not typical of subtype GS. Compared with the TCGA GC cohort, the tumor-mutation burden in this cohort was higher than that in the GS subtype. Furthermore, a high degree of gene amplification in the *RTK-Ras* pathway was observed in 45% of cases, including *KRAS* (19.4%), *FGFR2* (11.2%), *MET* (7.1%), *HER2* (5.1%), and *epidermal growth factor receptor* (*EGFR*, 4.1%), and the frequency of the amplification was higher than that of both diffuse-type GC (32.4%) and subtype GS (17.5%) in the TCGA cohort. These results suggest that the cohort might have unique features of the genetic variants. This novel approach did not only allow us to understand the notable genomic features of GC but also exhibited therapeutic targets.

## Immune checkpoint inhibitors for gastric cancer

Recently, ICIs that reactivate tumor-related T lymphocytes have been shown to be effective against various types of malignant tumors with their efficacy. GC is also treated with ICIs; however, the response rates are limited. Therefore, it is important to predict the anti-tumor effects of ICIs for GC and to select patients suitable for ICI treatment. For that purpose, in clinical practice, PD-L1 expression is often evaluated by immunohistochemistry (IHC) based on the tumor proportion score (TPS) and combined positive score (CPS) for indication [33, 34].

In the present case, unresectable or recurrent GC became a malignant disease, which is an indication for ICIs. The anti-tumor effect of nivolumab as a third-line drug or an increase in chemotherapy was reported in the ATTRACTION-2 study [35]. The utility of pembrolizumab as second-line or higher chemotherapy for MSI-high GC was also reported in the KEYNOTE-158 and KEYNOTE-061 studies [33, 36]. For various malignant tumors, including GC with MSI-high, the response rate and progression-free survival (PFS) of pembrolizumab were favorable in KEYNOTE-158. In KEYNOTE-061, pembrolizumab was significantly superior to paclitaxel monotherapy in the MSI-high subgroups. The frequency of MSI-high in GC is limited (6.3–21.9%), especially among Asians [37–39]. However, it is essential to select chemotherapy for the treatment of GC. More studies are needed to determine the clinical significance of MSI-high.

The utility of ICIs as a first-line chemotherapy was also evaluated. The KEYNOTE-062 study aimed to evaluate the anti-tumor activity of pembrolizumab, pembrolizumab plus chemotherapy (cisplatin plus fluorouracil or capecitabine), or chemotherapy alone in patients with untreated GC and GEJ adenocarcinoma with PD-L1 CPS of 1 or greater. Pembrolizumab was non-inferior to chemotherapy, with fewer adverse events [40]. Although the combination of PD-L1 inhibitor and chemotherapy was not superior to chemotherapy alone, pembrolizumab could be used as first-line chemotherapy for GC patients with a PD-L1 CPS of 1 or greater. To further improve the anti-tumor effect, biomarkers for extracting a more effective therapeutic target may be needed.

The CheckMate 649 trial evaluated PD-1 inhibitor-based first-line chemotherapy [34, 41]. Participants with unresectable tumors, PD-L1 CPS ≥ 5, non-HER2-positive GC, GEJ, or esophageal adenocarcinoma were classified into 3 groups and received nivolumab plus chemotherapy (oxaliplatin plus capecitabine or fluorouracil, leucovorin), nivolumab plus ipilimumab, or chemotherapy alone, respectively. After 24 months minimum follow-up, PD-1 inhibitor plus chemotherapy was superior to chemotherapy alone in overall survival (OS, median OS, 14.4 vs 11.1 months) and PFS (median PFS, 7.7 vs 6.0 months). The results revealed the utility of PD-1 for non-HER2-positive, PD-L1 CPS ≥ 5 in GC. Moreover, in all randomized patients, improvement of OS with nivolumab plus chemotherapy was significantly superior compared to chemotherapy alone (median OS, 13.8 vs 11.6 months). Currently, nivolumab plus cytotoxic chemotherapy is the standard first-line treatment for unresectable or recurrent GC. Meanwhile, nivolumab plus ipilimumab did not have a superior anti-tumor effect on OS compared to chemotherapy alone, although dual checkpoint inhibition has been proven to be effective in multiple solid tumors [42]. An increasing early death rate, a phenomenon known to be associated with immuno-oncology therapies [33, 40], was also observed in participants with GC treated with nivolumab plus ipilimumab. Meanwhile, the combination of PD-L1 and CTLA-4 therapy tended to prolong progression-free survival. Further research is needed to evaluate how tumor biology, molecular heterogeneity, TME, and other patient factors may affect the efficacy of the combined PD-L1 and CTLA-4 blockade. Genomics may help discover more detailed factors to predict the therapeutic effects of GC.

In the phase III KEYNOTE-811 trial, the anti-tumor effect of pembrolizumab for unresectable, HER2-positive GC or GEJ adenocarcinoma was evaluated [43]. Adding pembrolizumab to trastuzumab and cytotoxic chemotherapy induced complete responses in some participants and significantly improved the response rate compared with trastuzumab and cytotoxic chemotherapy only (74.4 vs 51.9%). In KEYNOTE-811, although 0.7% of the participants in the intention-to-treat population were known to have MSI-high tumors, 84.1% of participants had a PD-L1 CPS of one or more. It is interesting to determine whether the relative benefit of pembrolizumab plus trastuzumab and chemotherapy for OS is mainly associated with PD-L1 expression. Meanwhile, in KEYNOTE-062, the combination of pembrolizumab and cytotoxic chemotherapy was not superior to chemotherapy alone for GC patients with a PD-L1 CPS of 1 or more [40]. As pembrolizumab plus trastuzumab improved the response rate, combination therapy might be rational. In a previous study using patient-derived GC organoids, PD-L1 expression decreased in knockdown of *HER2* resulting in the inhibition of the *AKT-mTOR* pathway in PD-L1/HER2-positive GC cells and was correlated with an increase in cytotoxic T lymphocyte proliferation [44]. These results suggest that the co-expression of HER2 and PD-L1 may contribute to tumor cell immune evasion. This may explain why a combination of pembrolizumab and trastuzumab is favorable for GC treatment.

## Genomics for predicting the effect of immune checkpoint inhibitors on gastric cancer

MSI-high is a well-known marker for predicting the anti-tumor effect of ICIs for malignant tumors and has also been discovered via genome sequencing. Moreover, genomics revealed some surrogate markers related to the response rate of ICIs for malignant tumors, such as highly activated immune cells in the TME and intestinal flora [31]. The epigenomic changes were also evaluated, and the potential to predict the response to ICI therapy for GC was also shown [45, 46]. Genome-wide chromatin accessibility of circulating CD8 + T lymphocytes in the peripheral blood of patients was assessed using ATAC-seq. High chromatin openness at specific genomic positions of circulating CD8-positive T lymphocytes demonstrates a significantly better survival than closed chromatin [47].

In addition to the conventional genome sequence of cancer cells, it has become possible to analyze the antigen receptor repertoire of TILs, which makes it possible to capture an overview of acquired immunity to tumors more comprehensively. Substantial heterogeneity was observed in the TME between subtypes [48]. MSI-high and EBV-positive GC harbored many intense T cell infiltrates, a subtype of GS, half of which had tertiary lymphoid structures (TLS) enriched with CD4 + T cells, macrophages, and B cells. In contrast, most CIN GC exhibit T cell exclusion and infiltrating macrophages. Moreover, immune-poor CIN GCs were associated with *MYC* activity and *CCNE1* amplification, and these characteristics could be essential for predicting the anti-tumor effects of ICIs.

Recently, T cells, which are conventionally considered to play a major role in anti-tumor immunity, as well as B cells, which are responsible for humoral immunity, have attracted attention as they play an important role in tumor immunity. In a report on antigen receptor repertoire analysis of TILs in diffuse GC tissue, monoclonal B cells proliferated in many cases, and the major antigen of their antibody was sulfated glycosaminoglycan [49]. A relationship between ICIs and B cells has also been reported [50–52]. In malignant melanoma and renal cell carcinoma, the genes related to B cells were more enriched and expressed in the immune checkpoint inhibitor response group, and B cells infiltrated into the cancer tissue and the formation of TLS in the cancer tissue [50]. Interestingly, mature TLS may be related to the activation of T lymphocytes in tumor tissue.

## Precision medicine for gastric cancer

WGS revealed that GC could be classified into 4 subtypes with molecular pathology [10, 12, 13, 53], while each case had heterogeneity of the cancer genome and various patterns of gene variants. Clinical studies evaluating the combination of GC subtypes and therapeutic effects are needed to evaluate the efficacy of therapy for rare GC subtypes classified according to the patterns of genetic variants. Therefore, a designated study, called the master protocol, was established to evaluate therapeutic effects on each subgroup stratified by genetic profile [54]. The umbrella study was a master protocol to study multiple targeted therapies in the context of a single disease. Among 715 metastatic GC participants, the VIKTORY umbrella trial, a designated clinical study to evaluate standard chemotherapy and specific molecularly targeted therapy for each subgroup based on genetic variants and molecular marker expression in GC, was reported in 2018 [55] (Table 1). In the biomarker-specific trial, 105 participants were classified into 8 subgroups, including *RAS* (mutation or amplification)/*MEK* signature (high or low), *TP53* (mutation), *PIK3CA* (mutation or amplification), *MET* (amplification), *MET* (3 + by IHC), *TSC2* (null), *RICTOR* (amplification), and all negative; the efficiency of selective combination therapy consisted of inhibitors for each signaling pathway and anti-microtubule agent, respectively. As a result, in all subgroups, the OS of the groups that received selective therapy was significantly prolonged compared to that of the other group that received standard therapy (median 9.8 vs 6.9 months, *P* value < 0.0001). In particular, the greatest tumor-reduction effect was observed in the *MET* amplification subgroup treated with savolitinib, a MET inhibitor, compared to the other subgroups. Moreover, savolitinib was more effective among participants with frequent *MET* amplification in tumor tissue. The cell-free DNA (cfDNA) of *MET* is not only related to anti-tumor effects, but also to tumor progression relative to chemotherapy and recurrence. The results showed that liquid biopsy might also be useful in precision medicine. In 2021, personalized antibodies for gastroesophageal adenocarcinoma, a phase II umbrella study, were also reported [56] (Table 1). Participants were classified into 8 subgroups including immuno-oncology (PD-L1 CPS > 10, high microsatellite instability, tumor-mutation burden > 15 mutations per megabase, and/or EBV positivity), *HER2* amplification, *EGFR* amplification, *FGFR2* amplification, *MET* amplification, *MAPK/PIK3CA* aberrant, EGFR overexpressed, all negative, and received additional selective inhibitors for each signaling pathway, that is, nivolumab, trastuzumab, ABT-806 (EGFR inhibitor), bemarituzumab (FGFR2 inhibitor), none available (two participants excluded from intention to treat (ITT) received crizotinib, a MET inhibitor), ramucirumab (VEGFR2 inhibitor), ABT-806, and ramucirumab, respectively. The participants in each historical control group received only cytotoxic therapy. Of the 68 participants through ITT analysis, the 1-year survival rate was 66%, and the median OS was 15.7 months. First-line response rate (74%), disease control rate (99%), and median PFS (8.2 months) were superior to historical controls. These studies have reported relative improvements in patient outcomes so that additional specific molecularly targeted therapies on standard cytotoxic therapy may be useful for each GC subtype based on their genomic aberrations.

**Table 1:** Clinical studies for evaluating chemotherapy for gastric cancer with master protocols

Study name	VIKTORY	PANGEA
Year	2018	2021
Phase	III	II
Previous treatment	Presence (2nd-line)	Absence
Number of participants	715 (1st-line) 460 (2nd-line)	80
Number of participants with biomarker-driven treatment	105	68
Age, median (range, year)		61 (28–81)
Gender, male/female/NA		64/16
Subgroup		
RAS (mutation or amplification) or MEK signature (high or low)	25	–
TP53	25 (mutation)	–
PIK3CA	4 (mutation or amplification)	20 (MAPK/PIK3CA aberrant)
MET	24 (amplification)/4 (3 + by IHC)	–
TSC2	2 (null)	–
RICTOR	1 (amplification)	–
MSI-High	–	1
PD-L1	–	4 (CPS > = 10)
EBV positive	–	0
Tumor-mutation burden	–	0
HER2	–	16 (amplification)
EGFR	–	8 (amplification)/9 (overexpressed)
FGFR2	–	1 (amplification)
All negative	–	9
Historical control	266	12
Treatment		
RAS (mutation or amplification) or MEK signature (high or low)	Selumetinib + docetaxel	–
TP53	Adavosertib + paclitaxel	–
PIK3CA	Capivasertib + paclitaxel	Ramucirumab + mFOLFOX6
MET	Savolitinib, or savolitinib + docetaxel	mFOLFOX6 (none available)
TSC2	Vistusertib + paclitaxel	–
RICTOR	Vistusertib + paclitaxel	–
MSI-High	–	Nivolumab + mFOLFOX6
PD-L1	–	Nivolumab + mFOLFOX6
EBV positive	–	Nivolumab + mFOLFOX6
Tumor-mutation burden	–	Nivolumab + mFOLFOX6
HER2	–	Trastuzumab + mFOLFOX6
EGFR	–	ABT-806 + mFOLFOX6
FGFR2	–	Bemarituzumab + mFOLFOX6
All negative	–	Ramucirumab + mFOLFOX6
Historical control	Taxol/ramucirumab *(n* = 99), taxane-based (*n* = 105), irinotecan-based (*n* = 62)	mFOLFOX6
Progression-free survival Biomarker-specific vs. conventional (median)	5.7 months vs. 3.8 months	8.2 months vs. 6.7 months
Overall survival Biomarker-specific vs. conventional (median)	9.8 months vs. 6.9 months	15.7 months vs. 9.0 months

*MSI-H* microsatellite instability high, *CPS* combined positivity score, *EBV* Epstein–Barr virus, *TMB* tumor-mutation burden, *mFOLFOX* modified FOLFOX

## Utility of liquid biopsy for gastric cancer

Some studies have also revealed the utility of liquid biopsy in predicting the effect of treatment on GC. Various molecularly targeted therapies have also been tried for unresectable or recurrent GC; the anti-HER2 antibody trastuzumab was only indicated as first-line therapy for HER2-positive GC [57].

The addition of lapatinib, a tyrosine kinase inhibitor of the epidermal growth factor receptor and HER2, to capecitabine plus oxaliplatin did not increase OS in GC and GEJ participants with amplified HER2 [58]. However, the response rate of HER2-positive GC to trastuzumab is limited. Therefore, to predict the effect of trastuzumab on HER2-positive GC, liquid biopsy-based circulating tumor DNA (ctDNA), a cfDNA, was used [59]. As a result, somatic copy number alterations (SCNA) of the *HER2* gene were highly consistent with fluorescence in situ hybridization (FISH) data, and the *HER2* copy number decreased during treatment compared to baseline and PD levels because the *HER2* amplification clone was reduced via the anti-tumor effect of trastuzumab. Furthermore, patients with innate trastuzumab resistance presented high *HER2* SCNA levels during progression compared to baseline, while *HER2* SCNA decreased in patients with acquired resistance. Resistance-related tumor progression seems to interlock the persistence or recurrence of *HER2*-amplified copies in the blood. In addition, *NF1*, *PIK3CA/R1/C3*, and *HER2/ERBB4* mutations contributed substantially to resistance, while the *ERBB4* S774G mutation increased sensitivity to trastuzumab therapy. Moreover, the detected *HER2* SCNA level was better than the plasma carcinoembryonic antigen level for predicting tumor shrinkage and progression. These results show that ctDNA profiling may be useful for monitoring the occurrence and dissecting the potential molecular mechanisms of trastuzumab resistance. Considering the invasiveness of tissue acquisition in patients, the clinical and genetic evaluation of malignant lesions cannot be repeated. Liquid biopsies may provide informative data that can be obtained repeatedly, instead of tumor tissue analysis.

The efficiency of EGFR antibodies such as cetuximab and panitumumab for GC was also evaluated; however, there were no additional effects on the OS of GC participants [60, 61]. However, anti-EGFR antibody therapy may be effective in biomarker-selected populations. The REAL3 trial, a randomized first-line phase III clinical trial of chemotherapy or chemotherapy plus the anti-EGFR monoclonal antibody panitumumab, was performed to evaluate the anti-tumor effect of panitumumab for *EGFR*-amplified GC [62]. The copy number of *EGFR* was evaluated by either FISH or digital-droplet PCR in the pretreatment tissue and plasma cfDNA of the participants. Unfortunately, the addition of panitumumab to chemotherapy did not correlate with improved survival, even in participants with a significant *EGFR* copy number gain. Meanwhile, *EGFR* status could be reliably detected in tissue and cfDNA, and concordance between the two was observed in 95% of cases. According to these results, liquid biopsy of *EGFR* might be useful for selecting populations with high EGFR expression among GC patients. The study also revealed antagonistic effects between anti-EGFR agents and epirubicin, specifically in *EGFR*-amplified organoids, suggesting that EGFR inhibitors may not have to be used with anthracyclines for *EGFR*-amplified GC participants in future combinatorial trials.

In the TARGET study, analysis of both somatic mutations and copy number alterations across 641 cancer-associated gene panels in a single ctDNA assay for 100 participants with several malignant tumors was evaluated and compared to formalin-fixed paraffin-embedded tumor tissue analysis [63]. When a 2.5% variant allele frequency (VAF) threshold was applied, actionable mutations were identified in 41 of 100 participants, and 11 of them received matched therapy. However, comparisons evaluating the utility of ctDNA genotyping relative to tissue-based genotyping are lacking.

The SCRUM-Japan GI-SCREEN and GOZILA studies also revealed the utility of ctDNA in patients with advanced gastrointestinal carcinoma [64]. In GI-SCREEN, 5621 participants underwent tissue-based DNA sequencing, whereas 1687 participants underwent ctDNA-based sequencing in SCRUM-Japan GOZILA. To evaluate the utility of ctDNA profiling compared to that of tissue-based genotyping for trial enrollment among 287 patients for whom both tissue and ctDNA genotyping were performed, positive predictive values (PPV) of ctDNA were relative to clonality and VAF of ctDNA mutations if clonality was 0.3 or more, and PPV was above 80%. Moreover, ctDNA-based genotyping significantly shortened the screening duration (11 vs. 33 days, *P* values < 0.0001) and improved the trial enrollment rate (9.5 versus 4.1%, *P* values < 0.0001) without compromising treatment efficacy compared to tissue-based genotyping. In precision medicine, ctDNA is helpful for predicting treatment-effective populations based on genomic analysis.

## Genomics for metastatic lesions of gastric cancer

In therapeutic strategies for unresectable or recurrent malignant tumors, the target is not only the primary lesion but also metastatic lesions, and metastatic lesions are often resistant to various agents. For unresectable or recurrent GC, genetic variants and molecular expression of primary lesions are considered to determine the type of chemotherapy. In colorectal and breast adenocarcinomas, genetic variants of metastatic lesions are almost the same as those of primary lesions. In GC, a study revealed that the *HER2* expression status is almost the same between primary and metastatic lesions, whereby 30–50% of cases where differentiation is present between them is reported by the other. Generally, GC is a genomically heterogeneous malignant disease, and it is possible that there are discrepancies between the genetic variants of the primary and metastatic lesions. Pectasides et al. performed genomics for primary and metastatic lesions in several cohorts of GC participants and reported that there was a heterogeneous status of genetic variants between primary and metastatic lesions [65]. Comparing the mutation and amplification, respectively, between primary and metastatic lesions among 11 participants undergoing whole-exome sequencing, discrepancies of approximately 42% in genetic mutation and 63% in gene amplification status were present. In these cohorts, *TP53* mutations were almost homogeneous between primary and metastatic lesions in each case, whereas mutations in *PIK3CA* and amplification of *EGFR*, *HER2*, *CDK4/6*, and *MET* were heterogeneous between them. Seventy-five percent of the participants had heterogeneity of genetic variants associated with the tyrosine kinase receptor, especially, that the heterogeneity of *HER2* variant appears in 60% of cases. In 87.5% of cases that have heterogeneity of targeted genetic variants between primary and metastatic lesions, the results of the cfDNA analysis concur with those of the genetic analysis for metastatic lesions. Therefore, cfDNA reflects genetic variants in metastatic lesions; thus, some participants could receive adequate therapy based on cfDNA analysis. In clinical practice, genomics for metastatic lesions is not generally performed because additional tissue acquisition is required after diagnosis. However, if the genomic data of metastatic lesions could be obtained via analysis of cfDNA as a surrogate marker, GC patients might be able to receive adequate therapy and prolong their OS.

In the genomic and transcriptomic profiles, the differences between superficial primary tumor lesions and lymph node metastatic lesions are also shown [66]. In this study, the superficial and deep subregions of the primary tumor were annotated, and lymph node metastasis from GC resection specimens was assessed using genomic and transcriptomic profiling. As a result, IGF1, PIK3CD, and TGFB1 were overexpressed in the deep subregions and/or lymph node metastatic lesions, but not in the superficial subregions. Furthermore, 40% of mutations present in the deep subregions and/or lymph node metastatic lesions were not in the superficial subregions, although only 6% of mutations present in the superficial lesions were not in the deep subregions and/or lymph node metastatic lesions. This suggests that specimens obtained via endoscopic biopsies from primary GC lesions are not appropriate for genomic and transcriptomic profiling, although they are used for clinical diagnosis.

## Conclusion

Genomics has revealed the subtype of GC and the target of treatment for that, while immunological systems of the TME, such as TILs and TLSs, have been focused as biomarkers for predicting the anti-tumor effect via ICIs. Precision medicine has been used clinically and allows specific molecularly targeted therapy for each patient with GC to improve their prognosis. Meanwhile, genomic intratumor heterogeneity of GC exists, which may be related to the resistance to anti-tumor therapy. Therefore, genomic analysis of metastatic lesions, including liquid biopsy, is required to develop more appropriate therapeutic strategies. However, some cases, especially the subtype GS, are not necessarily adapted to previous chemotherapy, including cytotoxic therapy, molecularly targeted therapy, and ICIs. The genomic approach is expected to lead to the development of a new target, such as *RHOA* for subtype GS, to treat GC. Genomics is not only useful for identifying targets to treat GC, but also for screening and predicting the recurrence of GC. Furthermore, by monitoring genomic variants via liquid biopsy, it may be possible to select an appropriate treatment for GC with genomic alterations. Genomics has become an indispensable tool in clinical practice for GC and is expected to be further developed in the future.
